# Variations of retinal dysfunctions with the level of cannabis use in regular users: Toward a better understanding of cannabis use pathophysiology

**DOI:** 10.3389/fpsyt.2022.959347

**Published:** 2022-11-17

**Authors:** Thomas Schwitzer, Aldo Moreno-Zaragoza, Louis Dramé, Raymund Schwan, Karine Angioi-Duprez, Eliane Albuisson, Vincent Laprévote

**Affiliations:** ^1^Pôle Hospitalo-Universitaire de Psychiatrie d’Adultes et d’Addictologie du Grand Nancy, Centre Psychothérapique de Nancy, Laxou, France; ^2^INSERM U1254, Imagerie Adaptative Diagnostique et Interventionnelle, Université de Lorraine, Nancy, France; ^3^Faculté de Médecine, Université de Lorraine, Vandoeuvre-lès-Nancy, France; ^4^Fondation FondaMental, Créteil, France; ^5^Service d’Ophtalmologie, Centre Hospitalier Régional Universitaire de Nancy (CHRU Nancy), Nancy, France; ^6^CHRU-Nancy, Délégation à la Recherche Clinique et à l’Innovation, Département Méthodologie Promotion Investigation, Unité de Méthodologie, Data Management et Statistique, Unité de Méthodologie, Datamanagement et Statistiques, Nancy, France; ^7^Centre National de la Recherche Scientifique, Institut Élie-Cartan de Lorraine, UMR 7502, Vandoeuvre-lès-Nancy, France; ^8^Département du Grand Est de Recherche en Soins Primaires: DEGERESP, Nancy, France; ^9^INSERM U1114, Fédération de Médecine Translationnelle de Strasbourg, Département de Psychiatrie, Centre Hospitalier Régional Universitaire de Strasbourg, Strasbourg, France

**Keywords:** cannabis, retina, retinal function, electroretinogram, trait markers

## Abstract

The impact of regular cannabis use on retinal function has already been studied using flash (fERG) and pattern (PERG) electroretinogram. Delayed ganglion and bipolar cells responses were observed as showed by increased peak time of PERG N95 and fERG b-wave recorded in photopic condition. Hypoactivity of amacrine cells was also showed by decreased amplitudes of oscillatory potentials (OPs). However, it is unknown how these retinal anomalies evolve according to the level of cannabis use in cannabis users. The aim of this study was to longitudinally assess the retinal function during a treatment aiming to reduce cannabis use. We recorded PERG and fERG in 40 regular cannabis users receiving either an 8 weeks mindfulness-based relapse prevention program or an 8 weeks treatment-as-usual therapy. ERGs were recorded before treatment, at the end of it, and 4 weeks afterward. We found reduced peak times in PERG N95 and fERG b-wave (*p* = 0.032 and *p* = 0.024: Dunn’s *post-hoc* test) recorded at week 8 and increased amplitudes in OP2 and OP3 (*p* = 0.012 and *p* = 0.030: Dunn’s *post-hoc* test) recorded at week 12 in users with decreased cannabis use. These results support variations of retinal anomalies with the level of cannabis use, implying that reduction of cannabis use could restore retinal function in regular users.

## Introduction

The retina is nowadays accepted as a privileged easy-to access site to indirectly study human central synaptic transmission in psychiatric and addictive disorders (1–8). It is an anatomical and developmental extension of the central nervous system (CNS) due to its embryological origin (9). The retina is organized in layers of specialized neurons which are endowed with neurotransmission-signaling pathways, including glutamatergic and dopaminergic pathways (9). Thus, the study of retinal functioning offers the unique opportunity to investigate a complex neuronal network that shares several similar functional properties with the brain. The function of retinal neurons can be assessed objectively with electrophysiological techniques named electroretinogram (ERG), which have standardized protocols that allow reproducible results (10–13).

Our team has previously observed several retinal dysfunctions in regular cannabis users (14–20). Firstly, we showed a delay of approximately 6 ms in N95 wave peak time recorded with pattern electroretinogram (PERG) in regular cannabis users versus drug naive controls (17, 18). This result represents a delay in ganglion cell response, which may be linked with alterations in retinal glutamatergic neurotransmission. Secondly, we observed a significant delay of 1 ms in b-wave peak time recorded with light-adapted 3.0 flash ERG (fERG) in the same population (18). This represents a delay in bipolar cells response, implying that visual information is already delayed before ganglion cell processing. We also found significant decreased amplitudes in oscillatory potentials (OP) OP2 and OP3 as recorded with dark-adapted 3.0 fERG oscillatory potentials (15). These results suggest hypoactivity of retinal dopaminergic amacrine cells in regular cannabis users which may be associated with decreased retinal dopamine level (21). Using multifocal ERG (mfERG), we showed significant increase in peak times (+1 to 2 ms) for N2 (<2), N2 and P1 (2–5), P1 and N1 (5–10) and P1 (10–15) (19). This indicates a delay in the signal transmission of the cones system, located mainly in the central retina, in regular cannabis users compared to the healthy group. Our latest result showed significant increase in both the b-and the d-wave peak times recorded by On-Off ERG (20). These findings confirm that regular cannabis use impacts the post-receptoral cones pathway at the level of bipolar cells, affecting both the On and Off pathways.

However, we currently do not know how retinal anomalies evolve according to the level of cannabis use in cannabis users. In other words, it is not yet elucidated whether the variation of retinal anomalies pursues or not the evolution of cannabis consumption when it is reduced, enhanced, or stable. To this end, we longitudinally assessed in this study the retinal function during a treatment aiming to reduce cannabis use. We recorded PERG and fERG in 40 regular cannabis users attempting to modify their cannabis use with the help of either an 8 weeks mindfulness-based relapse prevention program or an 8 weeks treatment-as-usual therapy. Retinal function was assessed before treatment (week 0) at the end of the treatment (week 8) and 4 weeks after the end of the treatment (week 12).

The objective of this study was to longitudinally evaluate the retinal function using fERG and PERG in regular cannabis users before, at the end and after a period of treatment aiming to reduce cannabis consumption. Our hypothesis was that retinal anomalies evolve according to the level of cannabis use.

## Materials and methods

This study was performed to assess retinal function in regular cannabis users attempting to modify their cannabis use. It is an ancillary study of a prospective and monocentric open randomized controlled clinical study, called Macbeth (Méditation de pleine conscience et dépendance au cannabis: efficacité thérapeutique). The aim of Macbeth study was to assess the effect of a mindfulness-based relapse prevention (MBRP) program in regular cannabis users (22).

### Population and ethics statement

Regular cannabis users (*n* = 40) were recruited among the general population through a special press campaign in two phases: 20 subjects from February 18, 2019 to April 29, 2019 and 20 subjects from July 06, 2020 to August 08 2020.

Half of the regular cannabis users (*n* = 20) followed a MBRP program once a week during 8 weeks in two groups of 10, by two therapists who were formed in mindfulness practice, supervision and instruction based on the protocol defined in the MBRP treatment manual.

The other half (*n* = 20) followed a treatment as usual (TAU) once a week during 8 weeks. TAU consisted of a weekly individual interview with a psychiatrist-addictologist experienced in the care of cannabis users. This psychiatrist could choose the pharmacologic or the psychotherapeutic approach he considered relevant. Participants of both groups were then pooled for the analysis of retinal measurements.

Participants were informed about all the different details of the study. Written informed consent was obtained from all subjects. All participants received €50 in gift vouchers. The authors assert that all procedures contributing to this work comply with the ethical standards of the relevant national and institutional committees on human experimentation and with the Helsinki Declaration of 1975, as revised in 2008. All procedures involving human subjects were approved by the Ile de France Ethics Committee (num. 17-042).

### Inclusion criteria, clinical, and biological assessments

Eligible participants were adults aged 18–55 years. The inclusion criteria were regular cannabis users with an uptake of at least seven cannabis consumptions per week or with an abuse or a dependence diagnosis attested by the Mini-International Neuropsychiatric Interview (MINI) with at least 4 days per week of cannabis use or 8 days of consumption over the last 2 weeks. The cannabis consumptions were assessed with a TimeLine Follow Back (TLFB) and a positive urine toxicology screen for tetrahydrocannabinol (THC) metabolites was required. The history of cannabis use was assessed at the inclusion during an interview including the age of first cannabis use, total years of cannabis use, and average number of grams and joints smoked per week over the last month.

The non-inclusion criteria included an alcohol use disorder attested by the Alcohol Use Disorders Identification Test (AUDIT), other psychoactive substances use (except alcohol and tobacco), DSM-IV TR diagnosis of Axis I disorders (except anxious disorders) attested by the Mini International Neuropsychiatric Interview (MINI), history of neurological disease, an ongoing ophthalmological disease (except corrected refractive disorders) evaluated by visual acuity and fundoscopic examination and pregnancy or breastfeeding.

Electroretinogram measurements named PERG and fERG electroretinogram were performed at the inclusion, at week 8 and at week 12. Cannabis users filled TimeLine Follow Back (TLFB) attesting consumption of the seven previous days, once a week during 12 weeks, from inclusion to W12.

### Experimental protocol and analysis

Pattern electroretinogram and fERG were performed in accordance with International Society for Clinical Electrophysiology of Vision (ISCEV) standards (10, 12). The MonPackOne system (Metrovision, Pérenchies, France) was used for stimulation, recording and analysis. Electrical signals were recorded simultaneously from both eyes. Averaged retinal responses were first obtained from each eye, and then the values for given parameters (peak time and amplitude) were averaged over both eyes to allow analysis. Electrical signals were recorded on non-dilated (PERG) and dilated pupils (fERG, Tropicamide 0.5%) using Dawson, Trick, and Litzkow (DTL) electrodes (Metrovision, Pérenchies, France) placed at the bottom of the conjunctival sac. Pupil sizes were noted before fERG recordings, and remained systematically constant throughout the testing period. Ground and reference electrodes were attached to the forehead and external canthi.

For PERG, a black and white contrast reversible checkerboard, with 0.8 check size, 93.3% contrast level, 100 cd/m^2^ constant luminance white area, and four reversals per second was used. The participants were positioned 1 m from the screen. In the case of participants with refractive disorders, an appropriate optic correction was provided. At least 220 responses were recorded for each participant, with constant ambient room-lighting to achieve the best signal-to-noise ratio. Flash ERG recordings were performed in dark and light conditions. Participants were positioned 30 cm from the screen. They were dark-adapted for a period of 20 min before dark-adapted fERG were recorded. They were then light-adapted for 10 min to a light background set at 30 cd/m^2^ (cd/m^2^) managed by the MonPackONE system before light-adapted fERG was performed. At least eight and 16 responses, for dark- and light-adapted ERG, respectively, were recorded for each participant. Each retinal response is called according to the strength of the flash in candela.m^2^.s^–1^. To assess the functioning of the cone system, light-adapted 3.0 ERG was performed. To assess the functioning of the amacrine cells, flash ERG was performed under scotopic conditions with dark-adapted 3.0 oscillatory potentials. PERG and fERG data were analyzed with an ophthalmic monitor (Metrovision, Peìrenchies, France).

In this study, we considered ERG parameters previously assessed by our team that showed significant differences between regular cannabis users and healthy controls (15, 17, 18): N95 peak time, light-adapted 3.0 b-wave peak time and OP2 and OP3 amplitudes. N95 is believed to reflect the response of retinal ganglion cells. Light-adapted 3.0 b-wave is attributed to the retinal bipolar cells, postsynaptic to cone photoreceptors. Peak time of these waves [in milliseconds (ms)] denotes the time taken to reach the maximum amplitudes of these waves. OP2 and OP3 are known to reflect amacrine cells functioning. The OP2 and OP3 amplitudes [in microvolts (μV)] were measured from the trough of the preceding wave to the peak of the corresponding wave.

Protocols of ERG measurements and analysis of ERG recordings were previously described: for the PERG (17, 20) and for the fERG (15, 18, 20). Regular cannabis users underwent electroretinogram measurements-fERG and PERG-at three different recording times: week 0, week 8, and week 12. Week 0 correspond to the first recording time, performed at the inclusion, before treatment. A second recording took place at the eighth week, corresponding to the end of treatment. A third and last ERG recording was performed at week 12 of the study, 4 weeks after the end of treatment. We will refer to these different recording times as follow: inclusion as W0, eighth week as W8 and twelfth week as W12.

### Statistical analysis

Because of the non-parametric distribution of ERG parameters-N95 peak time, light-adapted 3.0 b-wave peak time, OP2 and OP3 amplitudes, non-parametric tests were used. We used-when appropriate to compare the independent groups–the Mann-Whitney U test or the Kruskal-Wallis test (one-way ANOVA on ranks) with *Post-hoc* comparison (Dunn’s *post-hoc* test). The level of significance is α = 0.05. Statistical analyses were performed using IBM-SPSS Statistics 22.0 (IBM corps).

## Results

### Demographic and substance use characteristics

The demographic and substance use characteristics of the participants at the inclusion are summarized in Table 1. At the inclusion, 40 cannabis users were included in this study (Table 3). At week 8, 14 cannabis users were excluded because they were lost to follow-up (Table 3). At week 12, 15 cannabis users were excluded because they were lost to follow-up (Table 3). Table 2 summarizes level of cannabis use at inclusion, week 8 and week 12, and difference between inclusion–week 8, week 8–week 12, and inclusion–week 12 in cannabis users. We divided cannabis users into three different groups based on the evolution of their cannabis consumptions–number of joints/week–between different recording times W0, W8, and W12: cannabis users with decreased consumption, cannabis users with stationary consumption and cannabis users with enhanced consumption (Table 3).

**TABLE 1:** Demographic and substance use characteristics of the participants at the inclusion.

	Cannabis users (*n* = 40)
Gender (male/female)^a^	24/16
Age (years)^b^	37 (27–40)
Education (years)^b^	13 (11–14)
Average number of alcohol uses/week^b^	4 (0–6)
Alcohol Use Disorders Identification Test (AUDIT) scores^b^	6 (3–8)
Fagerström test scores^b^	2 (0–4)
Average number of cigarettes/day^b^	4 (1–10)
Average number of pack-year of cigarettes^b^	4 (1–10)
Age of first cannabis use^b^	17 (15–19)
Total years of cannabis use^b^	18,5 (9–22)
Average number of joints/week^b^	25 (14–35)
Cannabis Abuse Screening Test (CAST) scores^b^	5 (4–5)
Average number of grams of cannabis/week^b^	5 (2–9)

a=Categorical variable represented as number.

b=Quantitative variable represented as median and interquartile range.

**TABLE 2:** Levels of cannabis use at inclusion, week eight and week twelve and differences: Inclusion–week 8, week 8–week 12, and inclusion–week 12.

	Median (IQR)
Number of joints per week at inclusion (*n* = 40)	25.0 (14.0:35.0)
Number of joints per week at 8 week (*n* = 26)	13.5 (5.0:23.2)
Number of joints per week at 12 week (*n* = 25)	21.0 (9.0:29.5)
Number of joints per week: difference between inclusion and week 8 (*N* = 26)	−9.0 (−18.5:−0.8)
Number of joints per week: difference between week 8 and week 12 (*N* = 23)	2.0 (−2.0:5.0)
Number of joints per week: difference between inclusion and week 12 (*N* = 25)	−10.0 (−18.0:−0.5)

**TABLE 3:** Number of cannabis users with decreased, enhanced or stationary consumption between W0–W8, W8–W12 and W0–W12.

	Inclusion	From inclusion to W8	From W8 to W12	From inclusion to W12
Cannabis users with decreased consumption	–	20	7	19
Cannabis users with stationary consumption	–	3	3	0
Cannabis users with enhanced consumption	–	3	13	6
Cannabis users lost to follow-up	–	14	17	15
Total (not missing)	40	26	23	25
Total	40	40	40	40

### Electroretinogram parameters

For the following analyses, N95 peak time, light-adapted 3.0 b-wave peak time, OP2 and OP3 amplitudes were considered because they represent retinal anomalies previously found in regular cannabis users (15, 17, 18). We present ERG parameters recorded at the end of each period such as follow: ERG at W8 for the period W0–W8, ERG at W12 for the period W0–W12 and ERG at W12 for the period W8–W12. All results are summarized in Table 4. Only significant results are presented below.

**TABLE 4:** Electroretinogram (ERG) parameters of participants.

Variation of cannabis use between week 0 (W0) and week 8 (W8)				

	Cannabis users with decreased use (*n* = 20)	Cannabis users with enhanced use (*n* = 3)	Cannabis users with stationary use (*n* = 3)	*P*-values
N95 peak time at W8 (ms)	91.6 ms (85.6:97.6)	99.4 ms (87.6:–)	104.0 ms (99.4:–)	0.026
Light-adapted 3.0 b-wave peak time at W8 (ms)	32.6 ms (30.5:35.9)	36.3 ms (30.0:–)	37.2 ms (37.2:–)	0.017
Oscillatory potential 2 amplitude at W8 (μV)	32.3 μV (23.2:38.8)	38.2 μV (34.3:–)	37.1 μV (31.8:–)	0.267
Oscillatory potential 3 amplitude at W8 (μV)	−26.4 μV (−37.8:−23.1)	−34.8 μV (−47.5:–)	−33.2 μV (−39.5:–)	0.256

**Variation of cannabis use between week 8 (W8) and week 12 (W12)**				

	**Cannabis users with decreased use (*n* = 7)**	**Cannabis users with enhanced use (*n* = 13)**	**Cannabis users with stationary use (*n* = 3)**	***P*-values**

N95 peak time at W12 (ms)	97.8 ms (86.3:109.0)	95.6 ms (90.9:104.6)	102.7 ms (85.4:–)	0.996
Light-adapted 3.0 b-wave peak time at W12 (ms)	35.4 ms (30.5:36.3)	32.2 ms (30.5:36.8)	30.5 ms (29.6:–)	0.249
Oscillatory potential 2 amplitude at W12 (μV)	45.0 μV (38.9:63.3)	30.7 μV (26.3:36.4)	36.1 μV (28.7:–)	0.016
Oscillatory potential 3 amplitude at W12 (μV)	−45.6 μV (−60.4:−40.6)	−30.3 μV (−42.7:−25.4)	−30.2 μV (−40.8:–)	0.028

**Variation of cannabis use between week 0 (W0) and week 12 (W12)**				

	**Cannabis users with decreased use (*n* = 19)**	**Cannabis users with enhanced use (*n* = 6)**	**Cannabis users with stationary use (*n* = 0)**	***P*-values**

N95 peak time at W12 (ms)	91.1 ms (86.3:105.1)	101.7 ms (95.2:107.3)	–	0.121
Light-adapted 3.0 b-wave peak time at W12 (ms)	32.2 ms (30.5:36.3)	33.6 ms (29.4:37.7)	–	0.877
Oscillatory potential 2 amplitude at W12 (μV)	36.0 μV (30.5:48.0)	40.0 μV (26.3:47.3)	–	1.0
Oscillatory potential 3 amplitude at W12 (μV)	−40.6 μV (–46.9:–28.9)	−38.3 μV (−52.1:–26.3)	–	1.0

Mann-Whitney U test or Kruskal-Wallis test (one-way ANOVA on ranks) with *Post-hoc* comparison (Dunn’s *post-hoc* test).

**FIGURE 1:**
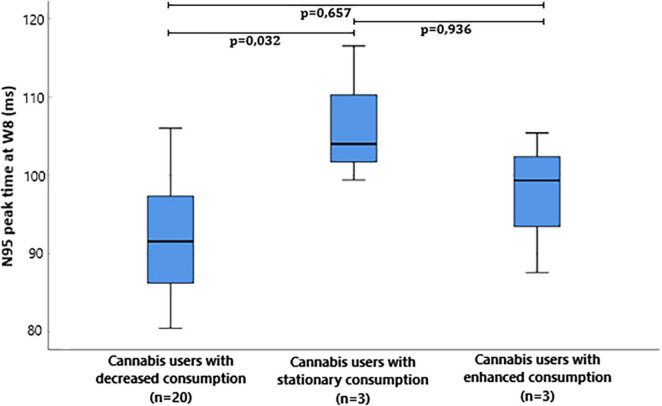
Box plot of Pattern electroretinogram (ERG) N95 peak time milliseconds (ms) at W8 in cannabis users divided in groups depending on the evolution of consumption between W0 and W8.

The median and interquartile range of the N95 peak time at W8 was 91,6 ms (85.6:97.6) in cannabis users with decreased consumption, 104.0 ms (99.4:–) in cannabis users with stationary consumption, and 99.4 ms (87.6:–) in cannabis users with enhanced consumption between W0 and W8 (Figure 1). Peak time of N95 at W8 was significantly different between the three groups (*p* = 0.026, Kruskal-Wallis test). *Post-hoc* comparison with Dunn’s *post-hoc* test showed that N95 peak time significantly differed between cannabis users with decreased consumption and cannabis users with stationary consumption (*p* < 0.032), but it failed to show any difference between cannabis users with decreased consumption and cannabis users with enhanced consumption (*p* = 0.657) and between cannabis users with enhanced consumption and cannabis users with stationary consumption (*p* = 0.936).

**FIGURE 2:**
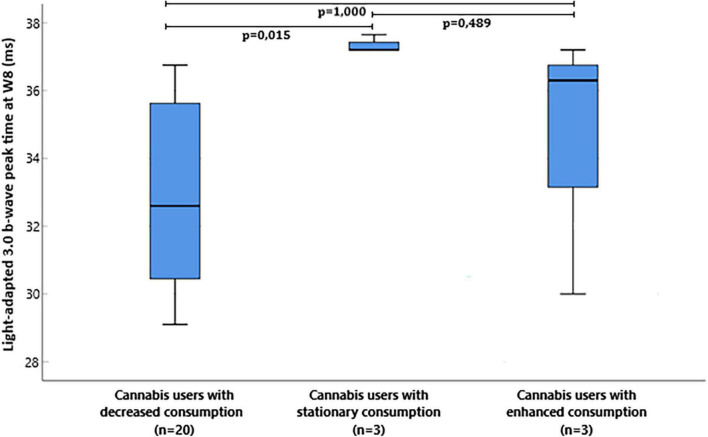
Box plot of flash electroretinogram (ERG) light-adapted 3.0 b-wave peak time milliseconds (ms) at W8 in cannabis users divided in groups depending on the evolution of consumption between W0 and W8.

The median and interquartile range of the light-adapted 3.0 b-wave peak time at W8 was 32.6 ms (30.5:35.9) in cannabis users with decreased consumption, 37.2 ms (37.2:–) in cannabis users with stationary consumption, and 36.3 ms (30.0:–) in cannabis users with enhanced consumption between W0 and W8 (Figure 2). Light-adapted 3.0 b-wave peak time at W8 was significantly different between the three groups (*p* = 0.017, Kruskal-Wallis test). *Post-hoc* comparison with Dunn’s *post-hoc* test showed that light-adapted 3.0 b-wave peak time significantly differed between cannabis users with decreased consumption and cannabis users with stationary consumption (*p* = 0.015), but it failed to show any difference between cannabis users with decreased consumption and cannabis users with enhanced consumption (*p* = 1.000) and between cannabis users with enhanced consumption and cannabis users with stationary consumption (*p* = 0.489).

**FIGURE 3:**
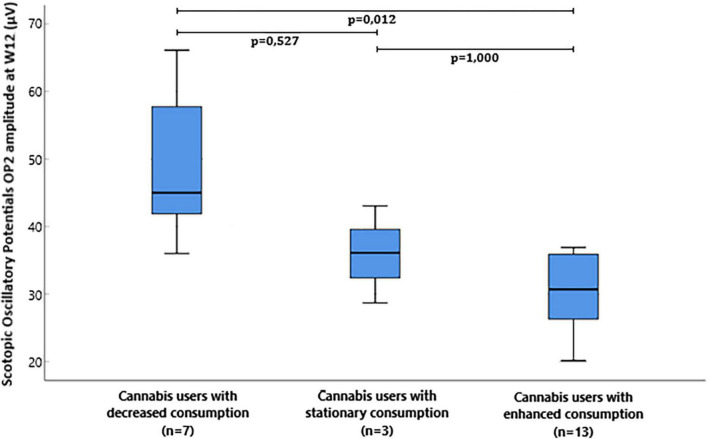
Box plot of flash electroretinogram (ERG) scotopic oscillatory potentials OP2 amplitude microvolts (μV) at W12 in cannabis users divided in groups depending on the evolution of consumption between W8 and W12.

The median and interquartile range of the OP2 amplitude at W12 was 45.0 μV (38.9: 63.3) in cannabis users with decreased consumption, 36.1 μV (28.7:–) in cannabis users with stationary consumption, and 30.7 μV (26.3:36.4) in cannabis users with enhanced consumption between W8 and W12 (Figure 3). OP2 amplitude at W12 was significantly different between the three groups (*p* = 0.016, Kruskal-Wallis test). *Post-hoc* comparison with Dunn’s *post-hoc* test showed that scotopic OP2 amplitude significantly differed between cannabis users with decreased consumption and cannabis users with enhanced consumption (*p* = 0.012), but it failed to show any difference between cannabis users with stationary consumption and cannabis users with enhanced consumption (*p* = 1.000) and between cannabis users with stationary consumption and cannabis users with decreased consumption (*p* = 0.527).

**FIGURE 4:**
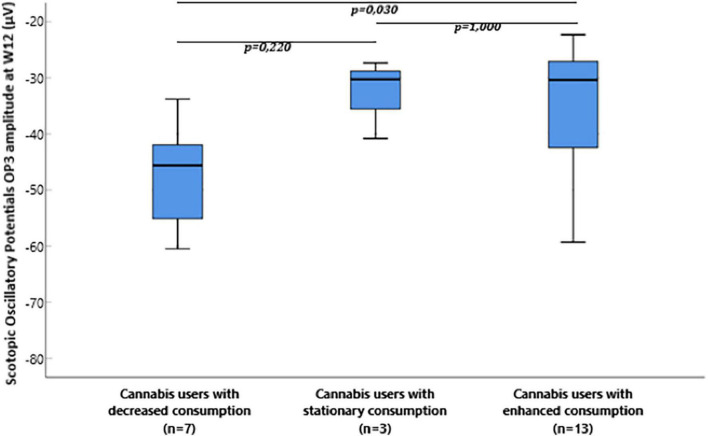
Box plot of flash electroretinogram (ERG) scotopic oscillatory potentials OP3 amplitude microvolts (μV) at W12 in cannabis users divided in groups depending on the evolution of consumption between W8 and W12.

The median and interquartile range of the OP3 amplitude at W12 was −45.6 μV (−60.4:−40.6) in cannabis users with decreased consumption, −30.2 μV (−40.8:–) in cannabis users with stationary consumption, and −30.3 μV (−42.7: −25.4) in cannabis users with enhanced consumption between W8 and W12. OP3 amplitude at W12 was significantly different between the three groups (*p* = 0.028, Kruskal-Wallis test). *Post-hoc* comparison with Dunn’s *post-hoc* test showed that scotopic OP3 amplitude significantly differed between cannabis users with decreased consumption and cannabis users with enhanced consumption (*p* = 0.030), but it failed to show any difference between cannabis users with stationary consumption and cannabis users with enhanced consumption (*p* = 1.000) and between cannabis users with stationary consumption and cannabis users with decreased consumption (*p* = 0.220) (Figure 4).

## Discussion

We found that retinal anomalies were modulated by the level of cannabis use in regular users. This modulation was showed by: one–reduced peak time in PERG N95 and fERG b-wave recorded at the end of the treatment–week 8–in users with decreased use in comparison with users with stationary use between week 0 and week 8; two–increased amplitude in OP2 and OP3 recorded at week 12 in users with decreased use in comparison with users with enhanced use between week 8 and week 12. According to these findings, retinal anomalies seem to regress with the reduction of cannabis consumption.

Previously, we observed an increase in peak time of N95 and light-adapted 3.0 b-wave and a decrease in OP2 and OP3 amplitude in regular cannabis users compared with controls (17, 18, 23). These results, respectively suggest a delay in the transmission of action potentials evoked by the retinal ganglion cells, a delay in the gradual variation of membrane potential in cone bipolar cells and a decrease in the overall response of amacrine cells. Here, these alterations seem to regress and return to a level close to normal functioning in cannabis users with decreased consumption of cannabis compared with cannabis users with stationary or enhanced consumption. This suggests that these anomalies appear reversible and not irreversible with a decrease use of cannabis. The results presented here support that the transmission of action potentials by the ganglion cells and the gradual variation of membrane potential in cone bipolar cells are carried out with a preserved speed in cannabis users with decreased consumption. Moreover, anomalies in peak times suggest that the total number of cells involved in the visual response is preserved, but that their functional properties are impaired. Besides, alterations in amplitudes support that the total number of cells involved in the response of retinal neurons is decreased. According to the results of this study, we suppose that both the quantitative and qualitative functional properties of retinal neurons–ganglion, cone bipolar and amacrine cells–are restored in cannabis users with decreased consumption.

Here, we showed that retinal anomalies varied with the level of cannabis use in regular users. Thus, we suppose that these variations are linked to the variations of retinal neurotransmitters level such as glutamate and dopamine (5, 24–27). Retinal neuron functioning is under the influence of several neurotransmitters such as glutamate and dopamine, to name a few (9). Regular cannabis use is known to induce modulations in brain glutamate and dopamine synaptic transmission (28–30). We previously supported that the increase in N95 peak time observed in regular cannabis users may be the consequence of modulation of retinal glutamatergic transmission, which is a well-known effect of regular cannabis use in the CNS (5). We also previously suggested that the decrease in OPs amplitudes observed in regular cannabis users was the consequence of reduced dopamine level, another well-known effect of regular cannabis use in the CNS (5). In this study, these retinal anomalies vary with the level of cannabis use, which probably impact the level of retinal neurotransmitters such as glutamate and dopamine. Interestingly, the retinal anomalies reduced as the level of cannabis use decreased. Thus, we suggest that the variations of retinal anomalies are linked to the retinal level of neurotransmitters. These hypotheses should be viewed cautiously since they are not objectified by measuring the precise level of neurotransmitters detected in the retina. However, these results are promising because the retina offers an indirect access to the functioning brain and could give biological markers of brain synaptic transmission dysfunctions in psychiatric and addictive disorders.

However, several potential mechanisms may be involved in the modulation of glutamatergic and dopaminergic transmission induced by cannabis use and at the origin of changes in neuronal function of the retina. Dopamine and glutamate are critical neurotransmitters involved in light-induced synaptic activity in the retina. Cannabinoid receptors CB1 and CB2 could have a crucial role since they are highly expressed in the neuronal layers of the retina (27, 31). Previous works found a correlation between endocannabinoid and dopaminergic system, thus supporting potential modulations of dopaminergic transmission in regular cannabis users (31). The effects of exocannabinoids on retinal functioning could also be mediated through glutamatergic receptors, especially NMDA-type glutamate receptors (NDMARs) since dopaminergic amacrine cells express functional NMDA receptors. Interestingly, NMDA receptors contribute to retrograde synaptic transmission from retinal ganglion cell photoreceptors to dopaminergic amacrine cells (32). In retinal neurons, dopamine-mediated dopamine D1 receptors stimulation provoked NMDARs hypofunction (33). The interaction between dopamine and glutamatergic function might impact on synaptic activity in retinal neurons.

Finding central biological markers of cannabis use is crucial since regular cannabis use is a major public health problem which is associated with severe CNS disorders (34, 35). Chronic exposure to cannabis is linked to cognitive impairments, especially in executive functions, memory and attention (34). Some of these cognitive impairments find restoration after cannabis withdrawal (34). Preclinical studies in animals also support the involvement of endocannabinoids and exocannabinoids in cognitive functions as well as the involvement of dopaminergic and glutamatergic transmission (36–39). Interestingly, retinal impairments seem to find restoration after cannabis use reduction as well. In the near future, it could be interesting to evaluate whether retinal electrophysiological measurements could be used as cognitive markers in regular cannabis users. They could facilitate the early detection and diagnosis of cognitive impairments in cannabis users. In order to confirm these hypotheses, future studies will record both retinal and cognitive functions in regular users.

This study has limitations and perspectives. Here, cannabis users could also have concomitant tobacco and alcohol use which can impact the effects on these substances on retinal function and central synaptic transmission (40, 41). Here, subgroups of cannabis users with decreased, enhanced or stationary use were small for some of them although the results presented here are consistent with previous results and promising. Futures studies should be performed with a larger number of participants to confirm the results presented here. The retinal abnormalities described in our study are functional impairments with underlying pathophysiological mechanisms that have not here been objectively identified. Thus, the precise molecular mechanisms underlying ERG anomalies should be investigated in specific studies. Molecular studies on retinal neurotransmission signaling pathways are crucial in future research in association with retinal electrophysiological measures. Here, only flash and pattern ERG were evaluated. It would be interesting in future studies to also evaluate multifocal ERG and On-Off ERG since they showed relevant results in previous studies. Finally, our groups of cannabis users had no history of psychiatric disorders. Since cannabis use is a risk factor leading to transition to psychiatric disorders such as schizophrenia or bipolar disorders, it would be interesting to longitudinally evaluate the retinal function in cannabis users who later develop or not a psychiatric disorder (42).

To conclude, these results support variations of retinal anomalies after changes in the level of cannabis use in regular cannabis users. The variations of retinal anomalies may be induced by changes in the level of retinal neurotransmitters such as dopamine or glutamate.

## Data availability statement

The raw data supporting the conclusions of this article will be made available by the authors, without undue reservation.

## Ethics statement

The studies involving human participants were reviewed and approved by the Ile de France Ethics Committee (num. 17-042). The patients/participants provided their written informed consent to participate in this study.

## Author contributions

All authors contributed to write the manuscript, concurred with the submission, and had approved the final manuscript.

## References

[1] Cosker E Schwan R Angioi-Duprez K Laprévote V Schwitzer T. New insights on the role of the retina in diagnostic and therapeutic strategies in major depressive disorder. *Neurosci Biobehav Rev.* (2020) 113:262–72. 10.1016/j.neubiorev.2020.03.006 32147530

[2] Komatsu H Onoguchi G Jerotic S Kanahara N Kakuto Y Ono T Retinal layers and associated clinical factors in schizophrenia spectrum disorders: a systematic review and meta-analysis. *Mol Psychiatry.* (2022). 10.1038/s41380-022-01591-x 35501407

[3] Lavoie J Maziade M Hébert M. The brain through the retina: the flash electroretinogram as a tool to investigate psychiatric disorders. *Prog Neuropsychopharmacol Biol Psychiatry.* (2014) 48:129–34. 10.1016/j.pnpbp.2013.09.020 24121062

[4] London A Benhar I Schwartz M. The retina as a window to the brain-from eye research to CNS disorders. *Nat Rev Neurol.* (2013) 9:44–53. 10.1038/nrneurol.2012.227 23165340

[5] Schwitzer T Schwan R Angioi-Duprez K Lalanne L Giersch A Laprevote V. Cannabis use and human retina: the path for the study of brain synaptic transmission dysfunctions in cannabis users. *Neurosci Biobehav Rev.* (2019) 106:11–22. 10.1016/j.neubiorev.2018.12.001 30773228

[6] Schwitzer T Schwan R Bubl E Lalanne L Angioi-Duprez K Laprevote V. Looking into the brain through the retinal ganglion cells in psychiatric disorders: a review of evidences. *Prog Neuropsychopharmacol Biol Psychiatry.* (2017) 76:155–62. 10.1016/j.pnpbp.2017.03.008 28336492

[7] Schwitzer T Lavoie J Giersch A Schwan R Laprevote V. The emerging field of retinal electrophysiological measurements in psychiatric research: a review of the findings and the perspectives in major depressive disorder. *J Psychiatr Res.* (2015) 70:113–20. 10.1016/j.jpsychires.2015.09.003 26424430

[8] Youssef P Nath S Chaimowitz GA Prat SS. Electroretinography in psychiatry: a systematic literature review. *Eur Psychiatry.* (2019) 62:97–106. 10.1016/j.eurpsy.2019.09.006 31553929

[9] Hoon M Okawa H Della Santina L Wong ROL. Functional architecture of the retina: development and disease. *Prog Retin Eye Res.* (2014) 42C:44–84. 10.1016/j.preteyeres.2014.06.003 24984227 PMC4134977

[10] Bach M Brigell MG Hawlina M Holder GE Johnson MA McCulloch DL ISCEV standard for clinical pattern electroretinography (PERG): 2012 update. *Doc Ophthalmol.* (2013) 126:1–7. 10.1007/s10633-012-9353-y 23073702

[11] Hoffmann MB Bach M Kondo M Li S Walker S Holopigian K ISCEV standard for clinical multifocal electroretinography (mfERG) (2021 update). *Doc Ophthalmol.* (2021) 142:5–16. 10.1007/s10633-020-09812-w 33492495 PMC7906932

[12] McCulloch DL Marmor MF Brigell MG Hamilton R Holder GE Tzekov R ISCEV standard for full-field clinical electroretinography (2015 update). *Doc Ophthalmol.* (2015) 130:1–12. 10.1007/s10633-014-9473-7 25502644

[13] Robson AG Nilsson J Li S Jalali S Fulton AB Tormene AP ISCEV guide to visual electrodiagnostic procedures. *Doc Ophthalmol.* (2018) 136:1–26. 10.1007/s10633-017-9621-y 29397523 PMC5811581

[14] Lucas A Thirion A Schwan R Krieg J Angioi-Duprez K Laprevote V Association between increased retinal background noise and co-occurrent regular cannabis and alcohol use. *Prog Neuropsychopharmacol Biol Psychiatry.* (2018) 89:335–40. 10.1016/j.pnpbp.2018.10.002 30292729

[15] Polli L Schwan R Albuisson E Malbos L Angioi-Duprez K Laprevote V Oscillatory potentials abnormalities in regular cannabis users: amacrine cells dysfunction as a marker of central dopaminergic modulation. *Prog Neuropsychopharmacol Biol Psychiatry.* (2021) 108:110083. 10.1016/j.pnpbp.2020.110083 32860840

[16] Schwitzer T Robert MP Giersch A Angioi-Duprez K Ingster-Moati I Pon-Monnier A Transient retinal dysfunctions after acute cannabis use. *Eur Addict Res.* (2016) 22:287–91. 10.1159/000446823 27376753

[17] Schwitzer T Schwan R Albuisson E Giersch A Lalanne L Angioi-Duprez K Association between regular cannabis use and ganglion cell dysfunction. *JAMA Ophthalmol.* (2017) 135:54–60. 10.1001/jamaophthalmol.2016.4761 27930757

[18] Schwitzer T Schwan R Angioi-Duprez K Giersch A Lalanne L Albuisson E Delayed bipolar and ganglion cells neuroretinal processing in regular cannabis users: the retina as a relevant site to investigate brain synaptic transmission dysfunctions. *J Psychiatr Res.* (2018) 103:75–82. 10.1016/j.jpsychires.2018.04.021 29783078

[19] Schwitzer T Henrion M-L Sarre D Albuisson E Angioi-Duprez K Giersch A Spatial localization of retinal anomalies in regular cannabis users: the relevance of the multifocal electroretinogram. *Schizophr Res.* (2020) 219:56–61. 10.1016/j.schres.2019.01.013 30696610

[20] Schwitzer T Schwan R Albuisson E Bugin C Zech G Angioi-Duprez K Delayed on- and off-retinal responses of cones pathways in regular cannabis users: an on-off flash electroretinogram case-control study. *J Psychiatr Res.* (2021) 136:312–8. 10.1016/j.jpsychires.2021.02.033 33636687

[21] Witkovsky P. Dopamine and retinal function. *Doc Ophthalmol.* (2004) 108:17–40. 15104164 10.1023/b:doop.0000019487.88486.0a

[22] Schneegans A Bourgognon F Albuisson E Schwan R Arfa M Polli L Mindfulness-based relapse prevention for cannabis regular users: preliminary outcomes of a randomized clinical trial. *Encephale.* (2021) 48:241–6. 10.1016/j.encep.2021.02.015 34092381

[23] Polli L Schwan R Albuisson E Malbos L Angioi-Duprez K Laprevote V Oscillatory potentials abnormalities in regular cannabis users: amacrine cells dysfunction as a marker of central dopaminergic modulation. *Prog Neuropsychopharmacol Biol Psychiatry.* (2020) 108:110083. 10.1016/j.pnpbp.2020.110083 32860840

[24] Laprevote V Schwitzer T Giersch A Schwan R. Flash electroretinogram and addictive disorders. *Prog Neuropsychopharmacol Biol Psychiatry.* (2015) 56:264. 10.1016/j.pnpbp.2014.04.005 24732440

[25] Schwitzer T Schwan R Angioi-Duprez K Giersch A Laprevote V. The endocannabinoid system in the retina: from physiology to practical and therapeutic applications. *Neural Plast.* (2016) 2016:2916732. 10.1155/2016/2916732 26881099 PMC4736597

[26] Schwitzer T Schwan R Angioi-Duprez K Ingster-Moati I Lalanne L Giersch A The cannabinoid system and visual processing: a review on experimental findings and clinical presumptions. *Eur Neuropsychopharmacol.* (2015) 25:100–12. 10.1016/j.euroneuro.2014.11.002 25482685

[27] Yazulla S. Endocannabinoids in the retina: from marijuana to neuroprotection. *Prog Retin Eye Res.* (2008) 27:501–26. 10.1016/j.preteyeres.2008.07.002 18725316 PMC2584875

[28] Bloomfield MAP Hindocha C Green SF Wall MB Lees R Petrilli K The neuropsychopharmacology of cannabis: a review of human imaging studies. *Pharmacol Ther.* (2019) 195:132–61. 10.1016/j.pharmthera.2018.10.006 30347211 PMC6416743

[29] Bloomfield MAP Ashok AH Volkow ND Howes OD. The effects of Δ9-tetrahydrocannabinol on the dopamine system. *Nature.* (2016) 539:369–77. 10.1038/nature20153 27853201 PMC5123717

[30] Colizzi M McGuire P Pertwee RG Bhattacharyya S. Effect of cannabis on glutamate signalling in the brain: a systematic review of human and animal evidence. *Neurosci Biobehav Rev.* (2016) 64:359–81. 10.1016/j.neubiorev.2016.03.010 26987641

[31] da Silva Sampaio L Kubrusly RCC Colli YP Trindade PP Ribeiro-Resende VT Einicker-Lamas M Cannabinoid receptor type 1 expression in the developing avian retina: morphological and functional correlation with the dopaminergic system. *Front Cell Neurosci.* (2018) 12:58. 10.3389/fncel.2018.00058 29662438 PMC5890097

[32] Liu L-L Spix NJ Zhang D-Q. NMDA receptors contribute to retrograde synaptic transmission from ganglion cell photoreceptors to dopaminergic amacrine cells. *Front Cell Neurosci.* (2017) 11:279. 10.3389/fncel.2017.00279 28959188 PMC5603656

[33] Socodato R Santiago FN Portugal CC Domith I Encarnação TG Loiola EC Dopamine promotes NMDA receptor hypofunction in the retina through D1 receptor-mediated Csk activation, Src inhibition and decrease of GluN2B phosphorylation. *Sci Rep.* (2017) 7:40912. 10.1038/srep40912 28098256 PMC5241882

[34] Broyd SJ van Hell HH Beale C Yücel M Solowij N. Acute and chronic effects of cannabinoids on human cognition-a systematic review. *Biol Psychiatry.* (2016) 79:557–67. 10.1016/j.biopsych.2015.12.002 26858214

[35] Meier MH Caspi A Ambler A Harrington H Houts R Keefe RSE Persistent cannabis users show neuropsychological decline from childhood to midlife. *Proc Natl Acad Sci U.S.A.* (2012) 109:E2657–64. 10.1073/pnas.1206820109 22927402 PMC3479587

[36] Brancato A Castelli V Lavanco G Marino RAM Cannizzaro C. In utero Δ9-tetrahydrocannabinol exposure confers vulnerability towards cognitive impairments and alcohol drinking in the adolescent offspring: is there a role for neuropeptide Y? *J Psychopharmacol.* (2020) 34:663–79. 10.1177/0269881120916135 32338122

[37] Brancato A Cavallaro A Lavanco G Plescia F Cannizzaro C. Reward-related limbic memory and stimulation of the cannabinoid system: an upgrade in value attribution? *J Psychopharmacol.* (2018) 32:204–14. 10.1177/0269881117725683 28880120

[38] Lavanco G Castelli V Brancato A Tringali G Plescia F Cannizzaro C. The endocannabinoid-alcohol crosstalk: recent advances on a bi-faceted target. *Clin Exp Pharmacol Physiol.* (2018). 10.1111/1440-1681.12967 29770478

[39] Oliveira da Cruz JF Busquets-Garcia A Zhao Z Varilh M Lavanco G Bellocchio L Specific hippocampal interneurons shape consolidation of recognition memory. *Cell Rep.* (2020) 32:108046. 10.1016/j.celrep.2020.108046 32814049 PMC7443618

[40] Dartois M Haudiquet N Albuisson E Angioi-Duprez K Schwan R Laprévote V Retinal dysfunctions in regular tobacco users: the retina as a window to the reward circuit in addictive disorders. *J Psychiatr Res.* (2021) 136:351–7. 10.1016/j.jpsychires.2021.02.023 33636691

[41] Kim JT Yun CM Kim S-W Oh J Huh K. The effects of alcohol on visual evoked potential and multifocal electroretinography. *J Korean Med Sci.* (2016) 31:783–9. 10.3346/jkms.2016.31.5.783 27134502 PMC4835606

[42] Schwitzer T Leboyer M Laprévote V Schwan R. Retinal electrophysiology and transition to psychiatric disorders in subjects under the influence of cannabis. *Prog Neuropsychopharmacol Biol Psychiatry.* (2022) 113:110466. 10.1016/j.pnpbp.2021.110466 34744025

